# H_2_S-Eluting Hydrogels Promote In Vitro Angiogenesis and Augment In Vivo Ischemic Wound Revascularization

**DOI:** 10.3390/biom14111350

**Published:** 2024-10-23

**Authors:** Joseph Giacolone, Robin Osofsky, Benjamin Matheson, Gabriela Perales, Reza Shekarriz, Nancy Kanagy, Ross M. Clark

**Affiliations:** 1 Department of Surgery, University of New Mexico, Albuquerque, NM 87131, USA; jgiacolone@salud.unm.edu (J.G.); rosofsky@gmail.com (R.O.); 2 Department of Cell Biology and Physiology, University of New Mexico, Albuquerque, NM 87131, USA; matheson.t.ben@gmail.com (B.M.); nkanagy@salud.unm.edu (N.K.); 3 Exhalix, LLC, Albuquerque, NM 87131, USA; reza@exhalix-llc.com

**Keywords:** wound healing, angiogenesis, hydrogen sulfide, tissue ischemia, therapeutics

## Abstract

Ischemic wounds are frequently encountered in clinical practice and may be related to ischemia secondary to diabetes, peripheral artery disease and other chronic conditions. Angiogenesis is critical to the resolution of ischemia. Hydrogen sulfide (H_2_S) is now recognized as an important factor in this process. H_2_S donors NaHS and GYY4137 were incorporated into the photosensitive polymer hydrogel gelatin methacrylate and evaluated. Human umbilical vein endothelial cell (HUVEC) culture was used to quantify toxicity and angiogenesis. Sprague Dawley rats were subjected to ischemic myocutaneous flap wound creation with and without application of H_2_S-eluting hydrogels. Tissue perfusion during wound healing was quantified using laser speckle contrast imaging, and gene and protein expression for VEGF were evaluated. Vascular density was assessed by CD31 immunohistochemistry. Successful incorporation of sulfide compounds was confirmed by scanning electron microscopy with energy-dispersive X-ray analysis, and under physiologic conditions, detectable H_2_S was present for up to 14 days by high-performance liquid chromatography. HUVECs exposed to hydrogels did not demonstrate excess cytotoxicity or apoptosis. A two-fold increase in angiogenic tube formation was observed in HUVECs exposed to H_2_S-eluting hydrogels. Rat ischemic flap wounds demonstrated greater perfusion at 14 days, and there was greater vascularity of healed wounds compared to untreated animals. A nearly two-fold increase in *VEGF* mRNA and a four-fold increase in VEGF protein expression were present in wounds from treated animals. Local-regional administration of H_2_S represents a novel potential therapeutic strategy to promote angiogenesis and improve wound healing after tissue injury or as a result of ischemic disease.

## 1. Introduction

Angiogenesis is a critical physiologic process that restores nutritive blood flow to ischemic tissues. Deficits in the angiogenic pathway have been implicated in obesity and diabetes, contributing to the development of chronic, non-healing wounds [1,2]. Understanding the mechanisms underlying new blood vessel growth is paramount in treating many common conditions, including peripheral artery disease (PAD), ischemic heart disease and poor wound healing. As the most common cause of non-traumatic limb amputation, PAD prevalence rises with age and affects 2 million elderly Americans, with a combined Medicare cost of USD 12 billion annually [1,2,3,4]. Chronic limb-threatening ischemia (CLTI) represents a severe form of PAD, and the prevalence of so-called “no option” CLTI—whereby surgical revascularization options are significantly limited—is rising [5]. Medical approaches to lower limb angiogenesis have largely failed to translate from laboratory studies to clinical practice in the past, leaving an opportunity for newly recognized candidate molecules to fill this role [6]. Novel therapeutic strategies are, therefore, necessary to address this common clinical condition.

Hydrogen sulfide (H_2_S), an endogenous gasotransmitter enzymatically produced by cystathione-ɣ-lyase (CSE), cystathionine-β-synthase (CBS) and 3-mercaptopyruvate sulfurtransferase (3-MST), has been previously shown by our group and others to be a powerful promoter of angiogenesis [7,8,9,10]. Diabetes and cardiovascular disease, common in aging populations, are associated with documented deficits in H_2_S levels [11,12,13]. Many effects of H_2_S are mediated through reversible protein sulfhydration of cysteine residues, which can either increase or decrease enzyme activity [14,15,16,17]. Hydrogen sulfide’s pro-angiogenic effect and short half-life make it an ideal target for a novel wound healing therapeutic. Recently, Yao et al. have shown that electrospun scaffolds containing H_2_S donors stimulate in vitro angiogenesis, work echoed by Han and colleagues who showed similar improvements in cell migration and proliferation as well as an ability to scavenge reactive oxygen species (ROS) with their sulfide-eluting electrospun mats [18,19].

While H_2_S holds promise as a novel therapeutic agent, major challenges related to drug delivery have been encountered. With a narrow therapeutic window to avoid toxicity, H_2_S has proven difficult to administer systemically beyond in vitro and early in vivo experiments [20,21,22]. Another concern with systemic administration is the potential for widespread, unmitigated angiogenic stimulation, which would be undesirable in the setting of occult malignancy, diabetic retinopathy or any other condition where uncontrolled angiogenesis would be unfavorable. To avoid these issues, strategies have been sought to deliver H_2_S compounds directly to desired tissues (local-regional delivery).

We describe a novel solution to this problem by utilizing a biocompatible matrix material previously used for drug delivery applications [23,24,25]. Gelatin methacrylate (GelMA) is a biodegradable photopolymer hydrogel that is liquid at room temperature, during which time it can be homogenously impregnated with therapeutic compounds [26]. After the introduction of sulfide compounds, GelMA is polymerized by exposure to 405 nm light, creating a semi-solid gel material. The hydrogel is relatively inert and can be easily introduced into in vitro and in vivo environments. GelMA-based H_2_S-eluting matrix material encapsulates known quantities of sulfide compounds in three-dimensional space, allowing tunable, controlled, long-lasting and localized delivery to desired tissues, resulting in effective augmentation of angiogenesis both in vitro and in vivo.

## 2. Methods

### 2.1. GelMA Preparation

GelMA hydrogels were prepared by aliquoting lithium phenyl-2,4, 6-trimethylbezoylphosphinate (LAP), corresponding to a concentration of 1% (*w*/*v*) into a light-protected 15 mL glass vial. Phosphate-buffered saline (PBS) solution (pH = 7.4) was then added to reach the desired volume and stirred at 60 °C for 20 min. Lyophilized GelMA (Cellink Biomaterials, Boston, MA, USA) was added to create a 10% *w*/*v* solution and stirred at 60 °C for 30 min until all material was dissolved.

NaHS (short-acting sulfide donor) and GYY4137 (long-acting sulfide donor) (Millipore Sigma, Burlington, MA, USA, #161527 and #SML2470) were used as sulfide donors and added to the prepared GelMA on a % weight/volume (% *w*/*v*) basis. The vial was vortexed for 30 s until the sulfide donor completely dissolved. Final prepared solutions were sterile-filtered using a 0.2 µm system. The GelMA solution was then evenly distributed across a custom-designed 3D-printed (poly)lactic acid micro-mold and exposed to 405 nm blue light for 5 min to completely polymerize the matrix. Prepared matrices were UV-sterilized as described below and sealed in a gas-tight vacuum-sealed packaging and stored at −20 °C until use.

### 2.2. Characterization of H_2_S Drug Elution

Standard volumes (100 µL) of donor-impregnated polymerized GelMA matrix material were deposited onto 1.5 mL microfuge tubes containing 1 mL of PBS. The tubes and PBS solution were deoxygenated in a sealed hypoxic glove box chamber purged with nitrogen to minimize oxygen exposure. Reaction tubes were incubated at 36 °C to simulate physiologic conditions. At pre-determined time points, samples of the PBS were collected after vortexing and subjected to reaction with monobromobibane (MBB) to form sulfide-dibibane (SDB) as described previously [27]. SDB concentration was quantified using fluorescence high-performance liquid chromatography (HPLC) (Agilent 1200 Series) on a Zorbax SB-C16 column (Agilent Technologies, Santa Clara, CA, USA) as described by Shen et al. [27]. Comparisons were made using a standard curve of known SDB concentrations (Dojindo Molecular Technologies, Rockville, MD, USA).

### 2.3. In Vitro Diffusion Assay

Diffusion assays were performed using methylene blue-eluting GelMA matrices embedded in 2% agarose gel. Serial, orthogonal digital photographs were obtained every 10 min for 24 h, and the diffusion rate and extent of diffusion from the matrix edge were quantified using planimetric analysis using Fiji Image J version 2.14 (National Institutes of Health, Bethesda, MD, USA) [28].

### 2.4. Scanning Electron Microscopy and X-Ray Diffusion Analysis

Surface microstructural characteristics and the elemental composition of plain and drug-laden matrices were assessed with scanning electron microscopy (SEM), and energy diffusion X-ray signatures (EDXas) were compared to known elemental samples.

### 2.5. Matrix Sterilization Microbiological Assays

GelMA matrices were prepared as described above and inoculated with non-pathogenic *E. coli* by incubation overnight in liquid media containing known organism concentrations of 1 × 10^6^ colony-forming units/mL. The matrices were then packaged in transparent vacuum-sealed envelopes, and ultraviolet (UV) energy (Stratagene Stratalinker 1800, La Jollla, CA, USA) was applied for 20 min per matrix side. Confirmation of sterilization efficacy was then tested by incubating the sterilized matrices in liquid culture media overnight and plating the media on standard agar. Plates were incubated for 7 days in physiologic conditions (37 °C), and qualitative analysis for organism growth was performed. Control matrices were treated identically, except no UV energy was applied.

### 2.6. In Vitro Cytotoxicity and Angiogenesis Assays

Cultured confluent human umbilical vein endothelial cells (HUVECs) were obtained from PromoCell (Heidelberg, Germany, Cat # 10171-910) and subjected to a range of concentrations of sulfide donors (NaHS and GYY4137) in a 96-well micro-assay. Matrix portions of 100 µL were incubated under physiologic conditions in cell culture media for 24 h, and the supernatant was added to the corresponding wells containing cultured cells. Cytotoxicity (cell membrane integrity) and apoptosis cascade activation (caspase 3/7) were quantified using process-specific fluorescence over 24 h (Incucyte, Sartorius, Gottingen, Germany). Fluorescence (Total Integrated Intensity) was quantified automatically using Incucyte software (https://www.sartorius.com/en) (Sartorius, Gottingen, Germany) and plotted over time. Statistical comparisons were made at 12 h of incubation with one-way ANOVA, specifying an α of 0.05.

Confluent HUVECs seeded onto collagen attachment factor were additionally subjected to an angiogenesis tube formation assay in the presence of a non-drug-laden matrix, 0.1% *w*/*v* NaHS, 0.2% *w*/*v* GYY4137 and VEGF (4 ng/mL) (Incucyte, Sartorius, Gottingen, Germany). Similar to the above, matrix portions of 100 µL were incubated under physiologic conditions in cell culture media for 24 h, and the supernatant was added to the corresponding wells containing cultured cells. Suramin 100 µM (angiogenic blocker) was used as a negative control. Drug concentrations were chosen based on results from cytotoxicity studies described above. Six biological replicate wells per group were investigated. High-resolution photographs of the entirety of each well during angiogenic tube formation were automatically taken each hour using Incucyte software. Maximal tube formation was noted in all groups after 14 h of growth, and this time point was chosen for analysis and comparisons. Images were analyzed by a blinded team member using an ImageJ Angiogenesis Analyzer to quantify the mean mesh size, which captures both the number of branch points as well as the tube length, as described previously [29].

### 2.7. In Vivo Wound Healing Model

Adult male Sprague Dawley rats (350–400 g, 10–12 weeks of age) were subjected to the creation of a 20 × 40 mm peninsular-shaped myocutaneous flap to model ischemic wound healing as described previously [30]. Briefly, a full-thickness tissue flap consisting of the epidermis, dermis and panniculus carnosus muscle was separated from the underlying paraspinal muscle and associated connective tissue, dividing perforating and radial vessels in the process, yielding a wound with progressive graded ischemia most apparent at the caudal (distal) flap and less severe at the cranial (proximal) portion. Treatment animals (n = 10) underwent surgical implantation of sterilized drug-eluting matrices matching the flap dimensions prior to flap wound closure. Matrices were placed between the elevated flap and the underlying wound bed and were not otherwise secured in place. Untreated animals (n = 10) underwent identical procedures but without implantation of matrices. Flap ischemia and subsequent revascularization were quantified using laser speckle contrast imaging (LSCI, FLPI Moor Instruments, Axminster, UK) of the flap area immediately pre- and postoperatively and on postoperative days 1, 3, 5, 7, 10 and 14. LSCI images were obtained at 25 Hz using a 1 sec time constant and a 4 ms exposure time. On postoperative day 14, the animals were humanely euthanized by pentobarbital overdose, and the healed flap wound was harvested en bloc for histologic, gene and protein expression assays.

### 2.8. Histologic Analysis and Immunohistochemistry

Healed flap wound tissue sections from both the proximal and distal aspects of the flap were paraffin-embedded after overnight fixation in IHC Zinc Fixative (BD Biosciences, Franklin Lakes, NJ, USA). Five-micron sections were subjected to staining with hematoxylin and eosin. Representative high-powered fields were photographed at 40× power (Nikon, Melville, NY, USA) after calibration with a stage micrometer. Using Image J, the cross-sectional thickness of the panniculus carnosus muscle was quantified (µm) and compared between groups.

Additional sections were subjected to immunohistochemical staining using the CD31 primary antibody at 1:50 dilution (mouse anti-rat BD Pharmingen #550300) and the biotinylated anti-Ig secondary antibody (BD Pharmingen HRP Kit #BDB551011, San Diego, CA, USA). DAB chromogen and dilute ammonia (0.2%) counterstaining permits slide analysis using light microscopy. Representative high-powered fields were photographed at 200× power and analyzed using Image J to quantify vascular density (CD31^+^ vessels/mm^2^) within engrafted ischemic distal flaps.

### 2.9. Western Blot Protein Expression Assays

Harvested flap tissue from the distal flap was collected after animal sacrifice and snap-frozen in liquid nitrogen. Protein concentrations were evaluated after tissue homogenization and lysis (Pierce BCA Protein Assay Kit, ThermoScientific #23225, Logan, UT, USA). Protein electrophoresis was carried out on 12% mini-protean TGX precast gels, followed by transfer onto PVDF Transfer Membrane (ThermoScientific #22860). Primary antibody to VEGF (Rabbit polyclonal, 1:1000 dilution, Novus Biologicals #NB100-2381, Denver, CO, USA) was incubated overnight at 4 °C, followed by secondary antibody (Bio-Rad Goat Anti-Rabbit IgG #12004162, Hercules, CA, USA) application for 1 h. GAPDH (Cell Signaling, Danvers, MA, USA, 1:1000 dilution, mouse primary antibody, #3700 with Bio-Rad Goat Anti-Mouse IgG secondary antibody, 1:2500 dilution, #12005867) served as the loading control. Blots were imaged on a Bio-Rad ChemiDoc System and analyzed using ImageJ Immunoblot Analyzer (Madison, WI, USA).

### 2.10. RT-PCR

Harvested healed flap tissue was preserved in RNALater at 4 °C until homogenization, and RNA was extracted using an RNEasy Fibrous Tissue Mini Kit (Qiagen #74704). Extracted RNA was converted to cDNA at concentrations of 1000 ng (Applied Biosystems High Capacity cDNA Reverse Transcription Kit, Waltham, MA, USA) on an automated thermal cycler. RT-PCR (Applied Biosystems 7500 Fast) was conducted in triplicate using Taqman (FAM) gene expression assays for VEGF (#Rn01511602_m1), CD68 (#Rn01495634_g1) and GAPDH (#Rn01775763_g1).

## 3. Results

### 3.1. Micro-molded Matrices Successfully Incorporate H_2_S Donor Molecules and Demonstrate Linear Diffusion Characteristics

**Figure 1:**
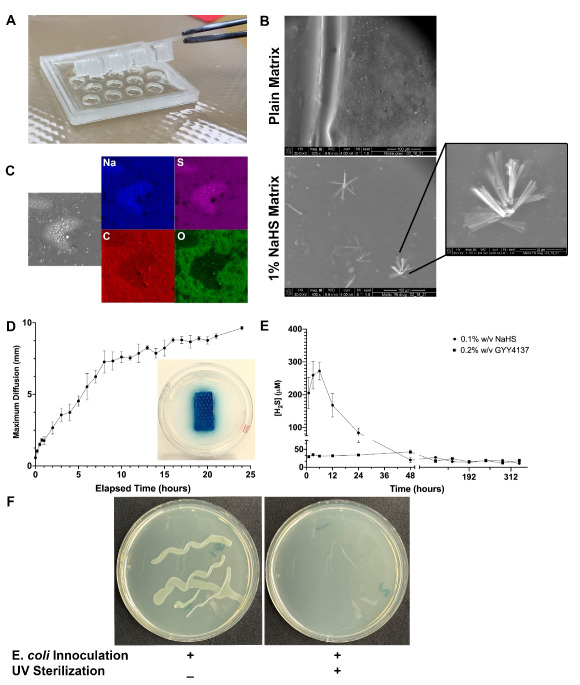
(**A**) Micro-molded gelatin methacrylate hydrogel matrix after photopolymerization. (**B**) Scanning electron microscopy of 1% *w*/*v* NaHS hydrogel demonstrating crystalline microstructures (lower panel and inset) embedded in an otherwise homogenous matrix background. Crystals are absent in plain matrix without drug (upper panel). (**C**) Energy diffusion X-ray analysis pseudocolor photomicrographs confirming sodium (Na) and sulfur (S) moieties present in drug-embedded matrix. Carbon (C) and oxygen (O) are not present. (**D**) Linear diffusion of methylene blue-eluting hydrogel matrix embedded in 2% agarose gel over 24 h with maximal diffusion 10 mm from the matrix edge. Inset: example dye-impregnated matrix during diffusion experiment. (**E**) High-performance liquid chromatography quantification of H_2_S concentrations measured from 0.1% *w*/*v* NaHS- and 0.2% *w*/*v* GYY4137-embedded matrices. NaHS demonstrates an early, high peak concentration with rapid fall, while GYY4137 demonstrates steady, long-term elution of sulfide. (**F**) Hydrogel matrices were successfully sterilized by application of UV energy with lack of growth of inoculated *E. coli* (right panel). Data are presented as mean ± standard error of the mean.

The 3D-printed micro-molding was successful in creating reproducible sulfide-laden matrices of tunable geometries (Figure 1A). SEM analysis of sulfide-impregnated matrix material revealed the presence of crystalline depositions, which were confirmed to be sulfide molecules with EDXas, whereas the plain matrix revealed a homogenous microstructure without crystalline depositions or sulfide moieties (Figure 1B,C). A methylene blue diffusion assay demonstrated a relatively linear diffusion rate of 0.375 mm/h over 24 h, with maximal diffusion observed approximately 10 mm from the matrix edge (Figure 1D).

### 3.2. Matrices Elute Bioavailable H_2_S with Tunable Dose Characteristics under Physiologic Conditions

Sulfide-eluting matrix material (NaHS 0.1% *w*/*v* and GYY4137 0.2% *w*/*v*) generated mean peak concentrations of 272 µM and 43 µM H_2_S, respectively, during simulated physiologic incubation. Peak concentration was noted at 8 h with NaHS before H_2_S levels were observed to fall precipitously. GYY4137 H_2_S concentrations persisted for up to 14 days, consistent with the slow hydrolytic nature of the molecule (Figure 1E).

### 3.3. Matrices Can Be Successfully Sterilized Using UV Energy

Application of UV energy to matrices inoculated with *E. coli* demonstrated an absence of growth after incubation in the culture medium, while matrices without UV energy application (control) demonstrated positive growth (Figure 1F).

### 3.4. H_2_S-Eluting Matrices Are Not Associated with Excess Cytotoxicity or Apoptosis Activation at Physiologically Relevant Donor Concentrations

**Figure 2:**
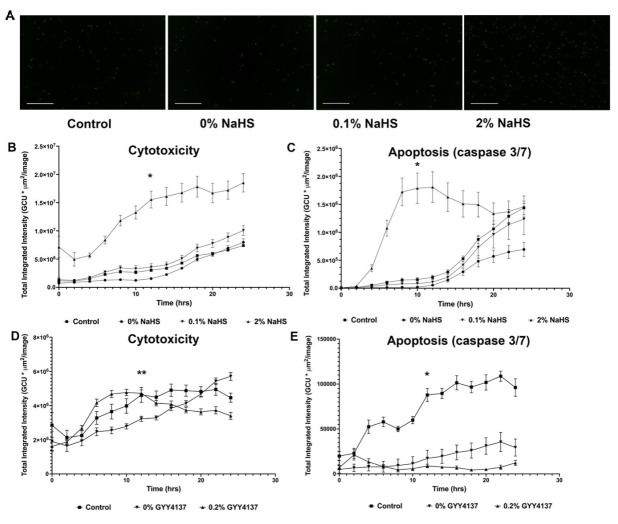
(**A**) Merged and fluorescent photomicrographs (40×) at 12 h during incubation, demonstrating no significant change in confluent human umbilical vein endothelial cell (HUVEC) cytotoxicity (loss of cell membrane integrity) with exposure to 0.1% *w*/*v* NaHS matrices, as compared to drug-free hydrogel matrix material (0% NaHS) and no matrix material (control). High dose (2% *w*/*v* NaHS) leads to widespread cytotoxicity at 12 h (*p* < 0.001), which continues for duration of experiment. (**B**) Similar findings at 12 h are observed when caspase 3/7 activation (apoptosis cascade) is measured by fluorescent marker (*p* < 0.001). (**C**) As shown, 0.2% *w*/*v* GYY4137-eluting matrices also lack excess cytotoxicity compared to plain matrix and control, though plain matrix was associated with mild protective effect at 12 h (*p* = 0.006). (**D**) In contrast, modest anti-apoptotic effect is observed with HUVEC exposure to both drug-free and GYY4137-eluting hydrogel matrices at 12 h (*p* < 0.001). (**E**) Data are presented as mean ± standard error of mean. All comparisons were made using one-way ANOVA at 12 h of incubation, α = 0.05, * *p* ≤ 0.001 and ** *p* ≤ 0.01. Scale bar is 300 µm. GCU = green average intensity.

HUVECs exposed to 0.1% NaHS- or 0.2% GYY4137-embedded matrices do not demonstrate excess cytotoxicity or apoptosis activation compared to controls (no matrix exposure and drug-free matrix material exposure) (Figure 2). Exposure to drug-free (0%) matrix material and 0.2% GYY4137-laden material demonstrated a modest anti-apoptotic effect, as evidenced by decreased caspase 3/7 activation (Figure 2E). Conversely, exposure to high-sulfide-concentration matrices (2% *w*/*v* NaHS) was associated with significant cytotoxicity and apoptosis cascade activation at nearly all time points (Figure 2A–C).

### 3.5. Sulfide-Eluting Matrices Induce Strong Angiogenic Tube Formation in Cultured Endothelial Cells

**Figure 3:**
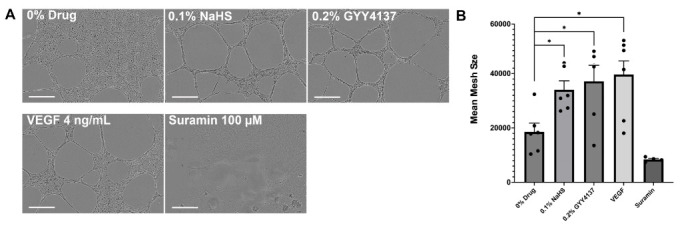
(**A**) Representative photomicrographs (40×) demonstrating response to human umbilical vein endothelial cells (HUVECs) and to H_2_S donor-embedded hydrogels at 14 h of incubation. Robust tube formation is observed on exposure to 0.1% *w*/*v* NaHS and 0.2% *w*/*v* GYY4137-eluting matrices as well as VEGF (4 ng/mL), as compared to control (0% drug) quantified by mean mesh size. (**B**) Suramin (100 µM), an angiogenic blocker, serves as negative control. Scale bar = 300 µm. Data are presented as mean ± standard error of the mean, * *p* ≤ 0.01, Student’s *t* test.

A 0.1% NaHS-embedded matrix increased tube formation in HUVECs at 14 h compared to non-sulfide-embedded matrices (Figure 3). As shown, 0.2% GYY4137- and VEGF-treated cells demonstrated similar levels of tube formation (all groups *p* < 0.01 compared to control). Suramin exposure was associated with poor angiogenic behavior as expected.

### 3.6. GYY4137-Eluting Matrices Improve Ischemic Wound Revascularization and Tissue Resilience to Ischemia

**Figure 4:**
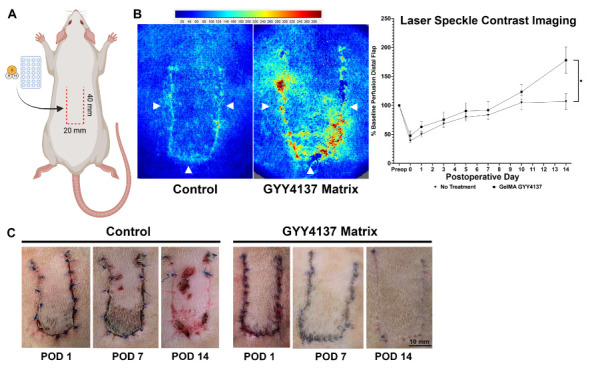
(**A**) Schematic of peninsular-shaped dorsal myocutaneous flaps (arrowheads) created in Sprague Dawley rats. Treated animals (n = 10) underwent implantation of H_2_S-eluting hydrogel matrices, while untreated (control) animals (n = 10) did not. (**B**) Laser speckle contrast imaging showing robust perfusion of flap wounds (incisions denoted by arrowheads) with treated animals (0.2% *w*/*v* GYY4137) achieving significantly greater perfusion (178% baseline) at 14 days compared to untreated animals (106% baseline). (**C**) High-resolution photographs showing progression of healing. Untreated flaps demonstrate ischemic ulceration of distal flap at postoperative days 7 and 14, while GYY4137-treated wounds heal without ulceration. Scale bar = 10 mm. Data are presented as mean ± standard error of mean, * *p* ≤ 0.01, Student’s *t* test.

Ischemic flaps of both GYY4137-treated and untreated animals demonstrated a mean decrement in distal perfusion of approximately 40% of baseline immediately after flap creation. During flap engraftment and revascularization, both treated and untreated animals demonstrated linear increases in perfusion. GYY4137-treated flaps ultimately achieved a mean perfusion 178% of baseline at 14 days compared to 106% in untreated flaps (*p* < 0.05) (Figure 4A,B). Planimetric photographs document improved visual wound healing in GYY4137-treated flaps as compared to untreated flaps, which demonstrate ischemic ulceration apparent at postoperative day 7 and extending to postoperative day 14 (Figure 4C).

**Figure 5:**
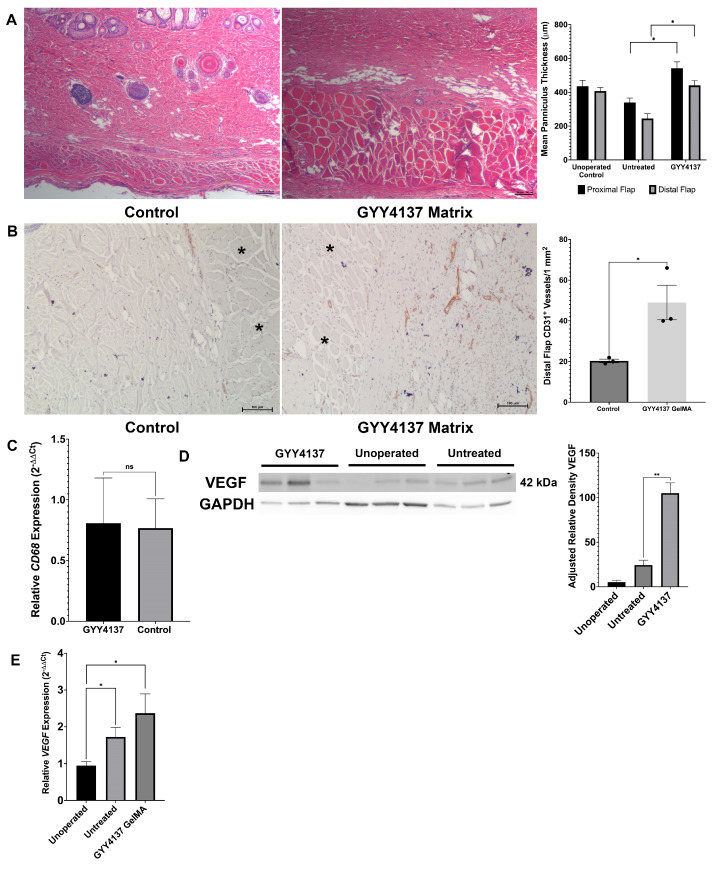
(**A**) Hematoxylin and eosin-stained histologic cross sections of healed distal flap wounds (200×), demonstrating preservation of the ischemia-sensitive panniculus carnosus muscle in GYY4137 hydrogel-treated animals. Scale bar = 100 µm. (**B**) CD31 immunohistochemistry (200×) of healed distal flap wound cross sections, demonstrating greater dermal and subcutaneous vessel count (brown stained structures) in GYY4137 hydrogel-treated animals. * indicates panniculus carnosus muscle. Scale bar = 100 µm. (**C**) GYY4137-treated flaps demonstrate no difference in macrophage marker *CD68* mRNA as compared to untreated animals, suggesting no persistent inflammatory response to the matrix. (**D**) Western immunoblot demonstrating four-fold upregulation of VEGF in sulfide-eluting hydrogel-treated distal flap wounds (n = 3) compared to untreated (n = 3) and unoperated (n = 3) animals. (**E**) H_2_S hydrogel-treated wounds are associated with upregulation of *VEGF* mRNA by RT-PCR. Data are presented as mean ± standard error of mean, * *p* ≤ 0.01, ** *p* ≤ 0.001, Student’s *t* test. Original Western blot images are available in Supplementary Materials.

Harvested distal flap wounds were observed to have predictable ischemia-related atrophy of the panniculus carnosus muscle layer in untreated animals (mean 245 µm distally compared to 407 µm in unoperated animals). Flaps treated with GYY4137 via matrix elution were noted to have preserved panniculus muscle thickness compared to untreated animals (mean 440 µm distal and 542 µm proximal, *p* < 0.05) (Figure 5A).

CD31 immunohistochemistry staining revealed greater vascularity within the dermis and panniculus carnosus muscle layers of distal flaps in GYY4137 matrix-treated animals as compared to untreated controls (49 vessels/mm^2^ compared to 20 vessels/mm^2^, *p* < 0.01) (Figure 5B).

### 3.7. GYY4137-Eluting Matrices Upregulate Angiogenic Gene and Protein Expression during Wound Healing

While untreated healed flap wounds were observed to have an expected upregulation of VEGF protein due to the angiogenic stimulus of tissue ischemia compared to unoperated animals, flap wounds exposed to GYY4137 demonstrated approximately four-fold greater VEGF protein expression compared to untreated flaps (*p* < 0.01) (Figure 5D). These data are consistent with mRNA expression of the *VEGF* gene, with untreated flaps demonstrating a 1.7-fold increase compared to unoperated animals and GYY4137-treated flaps observed to have a 2.4-fold increase (Figure 5E). Expression of the macrophage marker *CD68* by RT-PCR fails to demonstrate a difference between matrix-treated animals and untreated animals, suggesting that the observed VEGF expression was not due to persistent inflammatory response (Figure 5C).

## 4. Discussion

Gelatin methacrylate was successfully micro-molded and embedded with the sulfide-eluting compounds NaHS and GYY4137. To our knowledge, this is the first described application of GelMA specifically for the delivery of H_2_S compounds. GelMA’s unique property as a low-viscosity aqueous liquid prior to cross-linking allowed for uncomplicated introduction of each sulfide donor. Following the introduction of sulfide compounds, the now-solid GelMA was simple to handle and manipulate into in vitro and in vivo experimental applications after sterilization. Investigations into sulfide-embedded GelMA demonstrated that consistently measurable H_2_S could be detected eluting from the material, leading to significantly increased angiogenic behavior in tissue culture which was confirmed in small-animal mammalian wound healing.

Since the recognition of its pro-angiogenic properties, interest in techniques to deliver H_2_S as a therapeutic compound has increased dramatically in recent years. Wu and colleagues described utilizing a hyaluronic acid-based hydrogel combined with the H_2_S donor JK1 to influence wound macrophage polarization [31]. Others have recently reported on early work with electrospun scaffolds containing functional H_2_S donors with successful stimulation of angiogenesis both in tissue culture and in the chick chorioallantoic membrane assay [18]. Use of similar adaptations for wound dressings with self-catalytic H_2_S generation has also been shown to promote vessel ingrowth after excisional wounding in rats with attendant positive effects on oxidative stress [19]. Our work builds on these efforts by leveraging the advantages of GelMA, a versatile hydrogel with potential as a wound dressing component, combined with the powerful pro-angiogenic effects of sulfide agents [26].

Potential applications for local-regional sulfide therapies are broad and include promotion of wound revascularization and healing in settings of disadvantaged perfusion, such as diabetes-related micro- and macrovascular disease, peripheral artery disease leading to ischemic ulceration and tissue loss and trauma with attendant soft tissue disruption. Lower extremity wounds resulting from PAD with CLTI are generally addressed in a multidisciplinary fashion with surgical or percutaneous revascularization as the mainstay of therapy. Despite these efforts, a significant proportion of patients will go on to suffer major limb loss [32,33]. Additionally, the recognition of an increasing number of “no-option” CLTI scenarios, whereby anatomic or comorbidity constraints limit the application of traditional revascularization techniques, highlights a prime group of patients who may potentially benefit from the development of novel local-regional sulfide therapies [5].

While H_2_S and its related donor compounds certainly represent a promising novel therapeutic option for a variety of costly and debilitating clinical conditions, challenges remain in the practical development of clinic-ready technologies. The fleeting, toxic and volatile nature of H_2_S makes it particularly difficult to store and deliver to sites where its physiologic influence can be maximized. While no apparent morbidity was observed in the treated rats, off-target effects, such as unintended angiogenic stimulation in areas distant from the site of delivery, remain a risk that must be further investigated and mitigated for local-regional sulfide therapies to translate from the laboratory to the bedside.

## 5. Limitations

While we feel this work is an important contribution to the development of hydrogen sulfide-related therapeutics, which may one day provide clinical benefit, we acknowledge that there are several important limitations. First, our work leaves unanswered an important question related to the potential systemic spread of local-regionally delivered hydrogen sulfide compounds and future studies are needed to investigate the presence of sulfide in systemic circulation and in tissues remote from the site of administration. Additionally, while this work demonstrates the potential of local-regional hydrogen sulfide in the in vivo wound healing environment, it is not a rigorous evaluation of the effects of GelMA itself on mammalian tissues and additional work is needed to compare drug-laden matrices to sham matrices and also to define optimal storage conditions (shelf life, etc.) of candidate therapeutics. Additionally, while our results show promise for the use of H_2_S in ischemic wound healing, future studies should investigate the effects of the molecule under non-ischemic conditions, as a biologic effect may be present. Finally, as the importance of sex as a biological variable becomes more recognized, future work related to therapeutic development with hydrogen sulfide must include investigations related to differences in response to therapy in both male and female subjects.

## 6. Conclusions

Local-regional administration of sulfide donors represents a novel potential therapeutic strategy to promote angiogenesis and improve wound healing after tissue injury or as a result of ischemic disease. Delivery of sulfide compounds through these means should be further investigated for future translation to human applications.

## Data Availability

Data from this manuscript are available upon request.

## Supplementary Materials

The following supporting information can be downloaded at: https://www.mdpi.com/article/10.3390/biom14111350/s1, Original Western blot images.
