# Supplementary figures and images for: H2S-Eluting Hydrogels Promote In Vitro Angiogenesis and Augment In Vivo Ischemic Wound Revascularization
Source: Biomolecules. 2024 Oct 23;14(11):1350. doi: 10.3390/biom14111350 (PMC11591623; doi:10.3390/biom14111350)

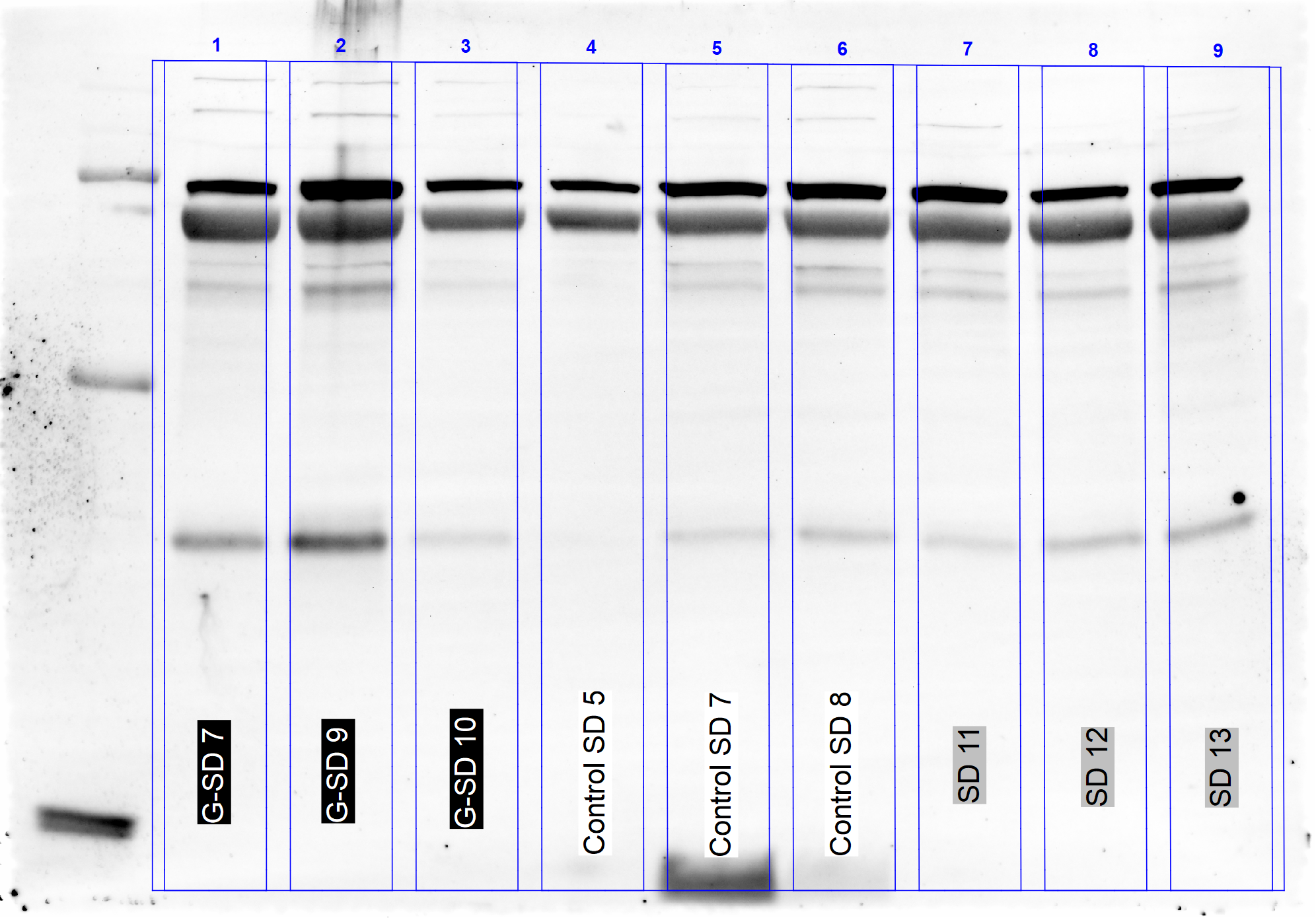

Supplement: Supplementary file 1 [file biomolecules-14-01350-s001.zip › biomolecules-3209882-original-images.tif]
